# Root water uptake and its pathways across the root: quantification at the cellular scale

**DOI:** 10.1038/s41598-019-49528-9

**Published:** 2019-09-10

**Authors:** Mohsen Zarebanadkouki, Pavel Trtik, Faisal Hayat, Andrea Carminati, Anders Kaestner

**Affiliations:** 1 Chair of Soil Physics, University of Bayreuth, Bayreuth, Germany; 2 Laboratory for Neutron Scattering and Imaging, Paul Scherrer Institut, Villigen, Switzerland

**Keywords:** Computational models, Plant cell biology

## Abstract

The pathways of water across root tissues and their relative contribution to plant water uptake remain debated. This is mainly due to technical challenges in measuring water flux non-invasively at the cellular scale under realistic conditions. We developed a new method to quantify water fluxes inside roots growing in soils. The method combines spatiotemporal quantification of deuterated water distribution imaged by rapid neutron tomography with an inverse simulation of water transport across root tissues. Using this non-invasive technique, we estimated for the first time the *in-situ* radial water fluxes [m s^−1^] in apoplastic and cell-to-cell pathways. The water flux in the apoplast of twelve days-old lupins (*Lupinus albus* L. cv. Feodora) was seventeen times faster than in the cell-to-cell pathway. Hence, the overall contribution of the apoplast in water flow [m^3^ s^−1^] across the cortex is, despite its small volume of 5%, as large as 57 ± 8% (Mean ± SD for n = 3) of the total water flow. This method is suitable to non-invasively measure the response of cellular scale root hydraulics and water fluxes to varying soil and climate conditions.

## Introduction

Roots serve a vital role (among other functions) in extracting water from the soil, transporting it to the shoot and sustaining transpiration. To fulfill this function, roots have a complex anatomical structure consisting of different cell layers with varying hydraulic conductivities. This composite structure offers different pathways for radial flow of water from the root surface towards xylem vessels^1–3^: (i) the apoplastic pathway through the intercellular space and the cell wall; (ii) the symplastic pathway via plasmodesmata channels extending across neighboring cells; and (iii) the transcellular pathway which involves crossing membranes of neighboring cells. Pathways (ii and iii) are commonly referred to as the cell-to-cell pathway.

Roots are capable of varying the permeability of their cells and tissues to fulfill multi-facet functions, such as (i) transport of water and nutrients towards the xylem vessels; (ii) protection against desiccation in drying soils; and (iii) avoidance of leakage of nutrients and photosynthesized compounds into the soil^4–6^. For instance, the permeabilities of the endodermis and exodermis are reduced by the deposition of Casparian bands and suberin lamellae^2,7,8^. These modifications are located perpendicular to the radial direction and external to the plasma membrane and reduce the permeability of the apoplastic pathway, i.e., they act as gatekeepers^9,10^. Another important factor affecting root permeability is the expression of aquaporins across the cell membrane^11–14^. Aquaporins are water channels across cell membranes and their expression and activity (opening and closure) affect the cell permeability and that of the whole root tissue.

The relative contribution of the apoplastic and cell-to-cell pathways to the total water flow across the root tissue is still a matter of debate^2,15,16^. The large size of intercellular space within the root cortex (ca. 100–320 µm) and the small width of primary cell walls (ca. 3–30 nm) suggest that the permeability of the apoplastic pathway should be much greater than that of the cell-to-cell pathway (via crossing the cell membrane or plasmodesmata)^17–19^. This argument suggests a prevalent contribution of the apoplastic pathway. An alternative argument, however, suggests that the apoplastic pathway partly impedes the flow of water^19–21^ because the apoplast has a small cross-sectional area (ca. 3% in maize) and it is tortuous. In addition, endodermis and exodermis interrupt the continuity of the apoplastic pathway, forcing water to flow through the cell-to-cell pathway. However, some studies claimed that the apoplastic barriers are imperfect and have a varying degree of interruption depending on plant species, developmental stages and growth condition^22–24^. Therefore, both pathways appear possible, but their relative contributions remain unanswered.

The relative importance of apoplastic and cell-to-cell pathways has been indirectly estimated based on measurements of root/cell hydraulic conductance using a root/cell pressure probe after inducing osmotically or hydrostatically driven water flow across the root tissue^25–27^. The idea was that hydrostatically driven flow does not distinguish between parallel apoplastic or cell-to-cell pathways - i.e. an increase in hydrostatic gradient equally increases the water flow through the apoplastic and cell-to-cell pathways. In contrast, an osmotically driven flow generates flow only across the cell-to-cell pathway, where selective membranes are involved. The importance of the two pathways on the total conductance of the root is estimated by comparing the two measurements. An alternative is to measure the hydraulic conductance of roots before and after blocking the apoplastic or the cell-to-cell pathways^15,28^. However, these methods are restricted to excised roots (invasive) and do not allow to resolve the local flow field of water across the tissue; for instance, even if the apoplast is completely blocked at the endodermis, it is not clear how much water flows through the apoplast inside the cortex. Direct identification and quantification of flow pathways require a method to measure the flow field of water across the root tissue under realistic conditions.

Motivated by the lack of experimental and non-invasive techniques, we developed an *in-situ* method to resolve the spatial distribution of water fluxes across the root tissue. The method consists of visualizing and quantifying the radial transport of deuterated water (D_2_O, as a tracer of normal water) inside the root of growing plant in soil using a rapid neutron tomography technique. In our previous works, we used neutron radiography to visualize in two-dimensions the transport of D_2_O inside the roots of plants growing in soil^29^. A diffusion-convection model including different pathways of water was developed to quantify the transport of water across the root tissue^30^. Relying on two-dimensional information, the radial flux of water was successfully quantified, but the model was not sensitive to the pathways of water across the root tissue. In other words, we could estimate the total flow of water entering the root in a given location, but we could not resolve the flow field across the root tissue and discriminate between apoplastic and cell-to-cell pathways. Recent advancements in neutron imaging allow for much faster scanning times^31–33^. When used for the water uptake investigations, Zarebanadkouki *et al*.^32^ performed on-the-fly neutron tomography with a scanning time of 180 seconds and Tötzke *et al*.^33^ used scanning time of 10 seconds, to successfully visualize D_2_O entering the root system in three-dimensions. However, it is not yet clear whether and how such information could be used to predict the pathways of water across the roots. Here, we tested: (i) whether series of rapid neutron tomographies (single tomography scanning time of 30 s and a voxel size of 45 µm) is capable of resolving gradients in D_2_O concentration along the radial distance to the root center, and (ii) whether we could inversely calculate the relative importance of the two pathways by using a model of D_2_O transport that explicitly considers the apoplastic and cell-to-cell pathways.

## Results

**Figure 1:**
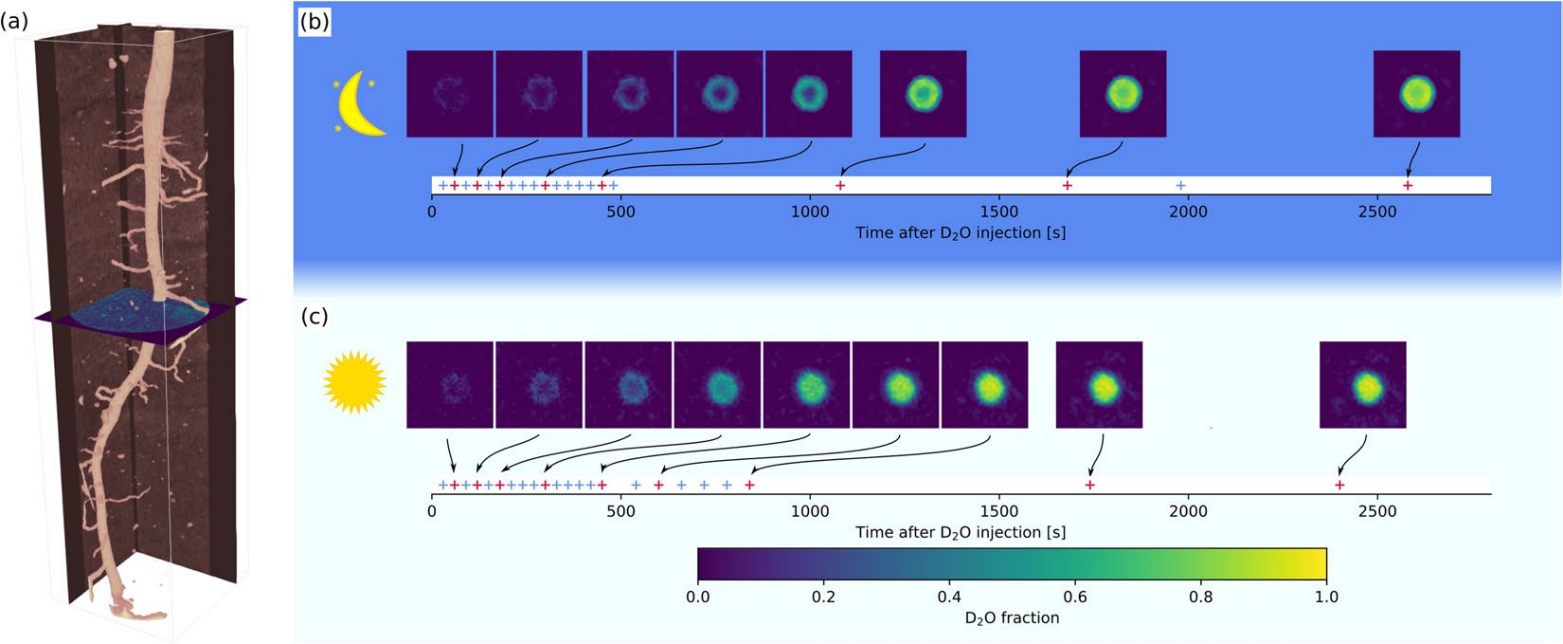
(**a**) Reconstructed root system of a twelve-days old lupine before D_2_O injection. D_2_O was injected in soil and its redistribution within soil and roots was monitored using time-series neutron radiography. (**b**,**c**) Transverse section of neutron tomographs showing the concentration of D_2_O (fraction of D_2_O to total water) across the taproot of two exemplary plants at different times after D_2_O injection during the night (**b**) and the daytime (**c**). The transverse sections are taken at a depth of 5 cm from the soil surface. The given time (t) refers to the time after D_2_O injection. These images show that the transport of D_2_O across the root tissue was faster during daytime than nighttime. During nighttime, D_2_O transport was significantly slowed down by the presence of endodermis. Detailed time-series neutron tomographs are shown as Supplementary Information Fig. S1. Note that these transverse sections show only the concentration of D_2_O in root and not the one in soil.

An exemplary reconstructed 3D root system of a twelve days old lupin before D_2_O injection is shown in Fig. 1a. This image was reconstructed from 180 projections uniformly distributed over angular views of 0 to 179 degrees. D_2_O was injected at the soil surface while the root system was simultaneously imaged at varying angles for a period of two hours. Exemplary transverse sections at a depth of 5 cm from the soil surface illustrate the transport of D_2_O across the root tissue of two plants during the night (negligible transpiration) and daytime (illuminated with constant light intensity) (Fig. 1b,c and Supplementary Information Fig. S1). The tomograms clearly resolved gradients in the concentration of D_2_O across the root tissue and showed a steep drop in D_2_O concentration at the endodermis. The gradients were steeper during nighttime than daytime indicating that the presence of an endodermis significantly limited the transport of D_2_O across root tissue at night, while its effect was less pronounced during daytime measurements.

**Figure 2:**
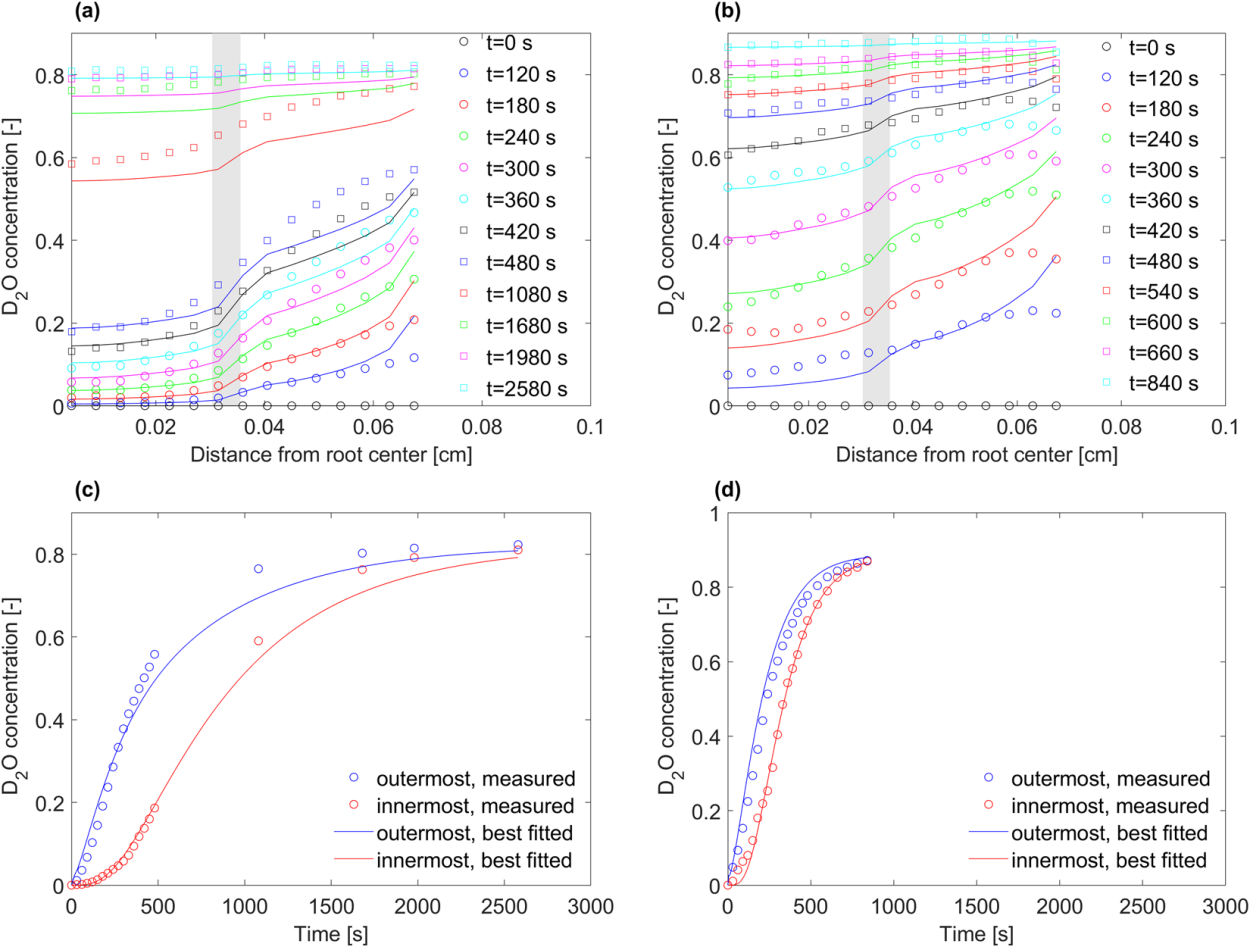
Exemplary profiles of D_2_O concentration (fraction of D_2_O to total water) as a function of distance from the root center at selected times after immersion in D_2_O during nighttime (**a**) and daytime (**b**). D_2_O concentration (fraction of D_2_O to total water) in the stele and root cortex as a function of time after immersion in D_2_O during nighttime (**c**) and daytime (**d**). Root stele and the root cortex represent the average concentration in the third innermost and third outermost voxels of the roots. In all figures, the circles show concentrations obtained from image processing of CTs and the lines show the best-simulated profiles of concentration. In (**a**,**c**) the region shown with a grey color indicates the position of endodermis.

We quantified the reconstructed neutron tomograms to calculate profile of D_2_O concentration across the root tissue (Fig. 2). The profiles showed steeper gradients in the concentration of D_2_O across the root tissue during nighttime while they were relatively flat shortly after D_2_O injection during the daytime. The concentration of D_2_O was averaged across regions with a size of three voxels at the outermost parts and innermost parts of roots (iso distance bands from the surface) to represent the average D_2_O concentration in the root cortex and the root stele, respectively (Fig. 2c,d). Our results showed: (i) a faster increase in the concentration of D_2_O in both root cortex and root stele during daytime than nighttime, and (ii) a steeper gradient in concentration of D_2_O between cortex and stele during nighttime compared to daytime.

**Figure 3:**
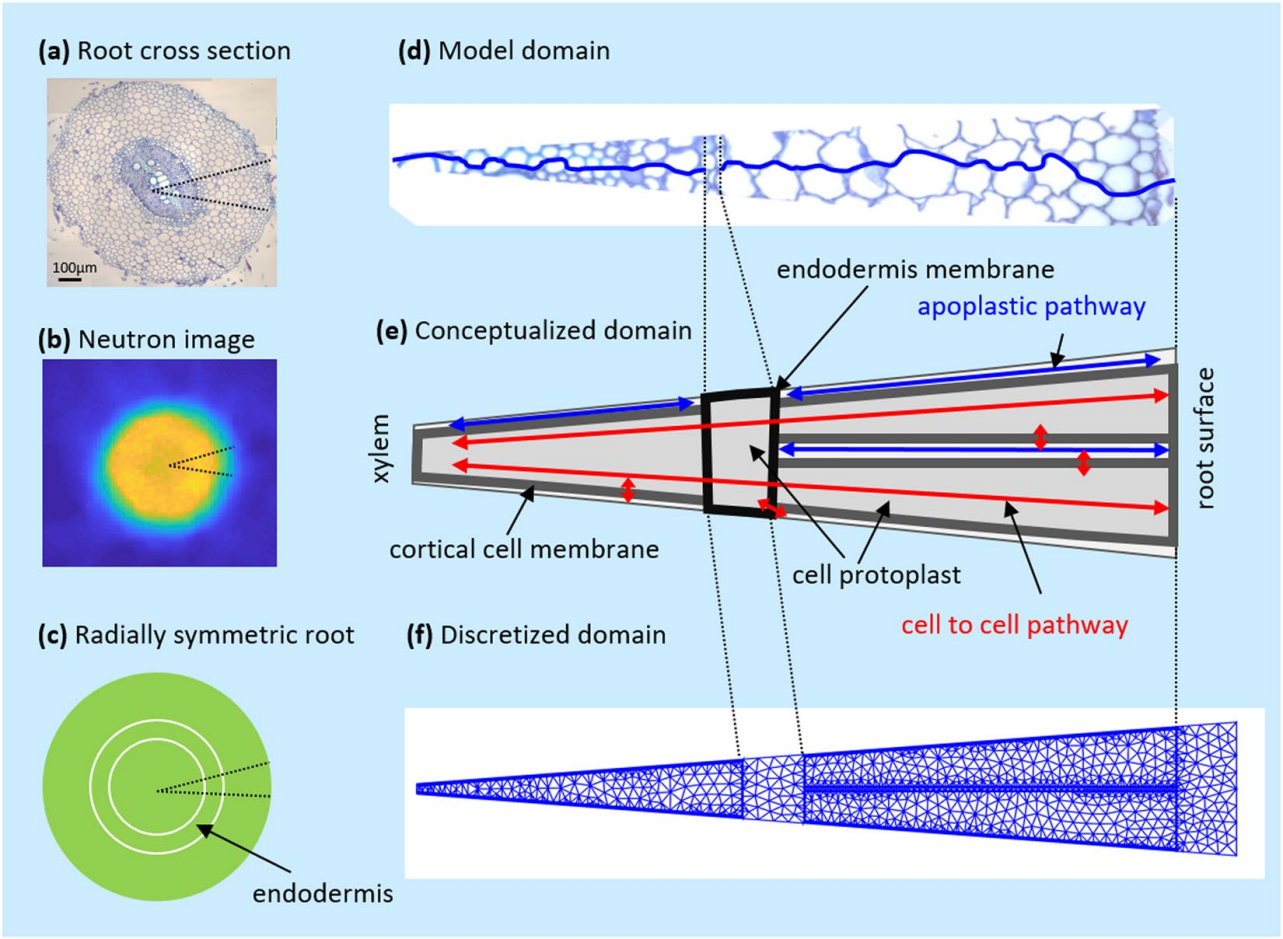
(**a**) Light transmission microscopy of a cross-section of the taproot of a twelve-days old lupin at a distance of 5 cm from the soil surface. (**b**) Neutron tomograms showing a cross-section of the taproot. The color is proportional to the water content. (**c**) Radially symmetric root schematization. (**d**) The fraction of the root tissue used to set-up the model with one apoplastic pathway indicated in blue. (**e**) Schematic of the flow domain across the root tissue. (**f**) Discretization of the flow domain into 2000 finite elements.

The transport of D_2_O into roots during the night was described by a diffusion equation and its transport during the day was described by a hydraulic model coupled with the convection-diffusion equation (Eq. 6). These equations were solved numerically for a conceptualized flow domain across the root tissue (Fig. 3). The model reproduced the measured profiles of D_2_O concentration during both nighttime and daytime (Fig. 2). We estimated the parameters (cell scaled diffusion coefficients and hydraulic conductivities of root tissue) from an independent inverse simulation of D_2_O transport measured in three plants during nighttime and three plants during daytime conditions (the parameters are given as Supplementary Information in Fig. S2).

**Figure 4:**
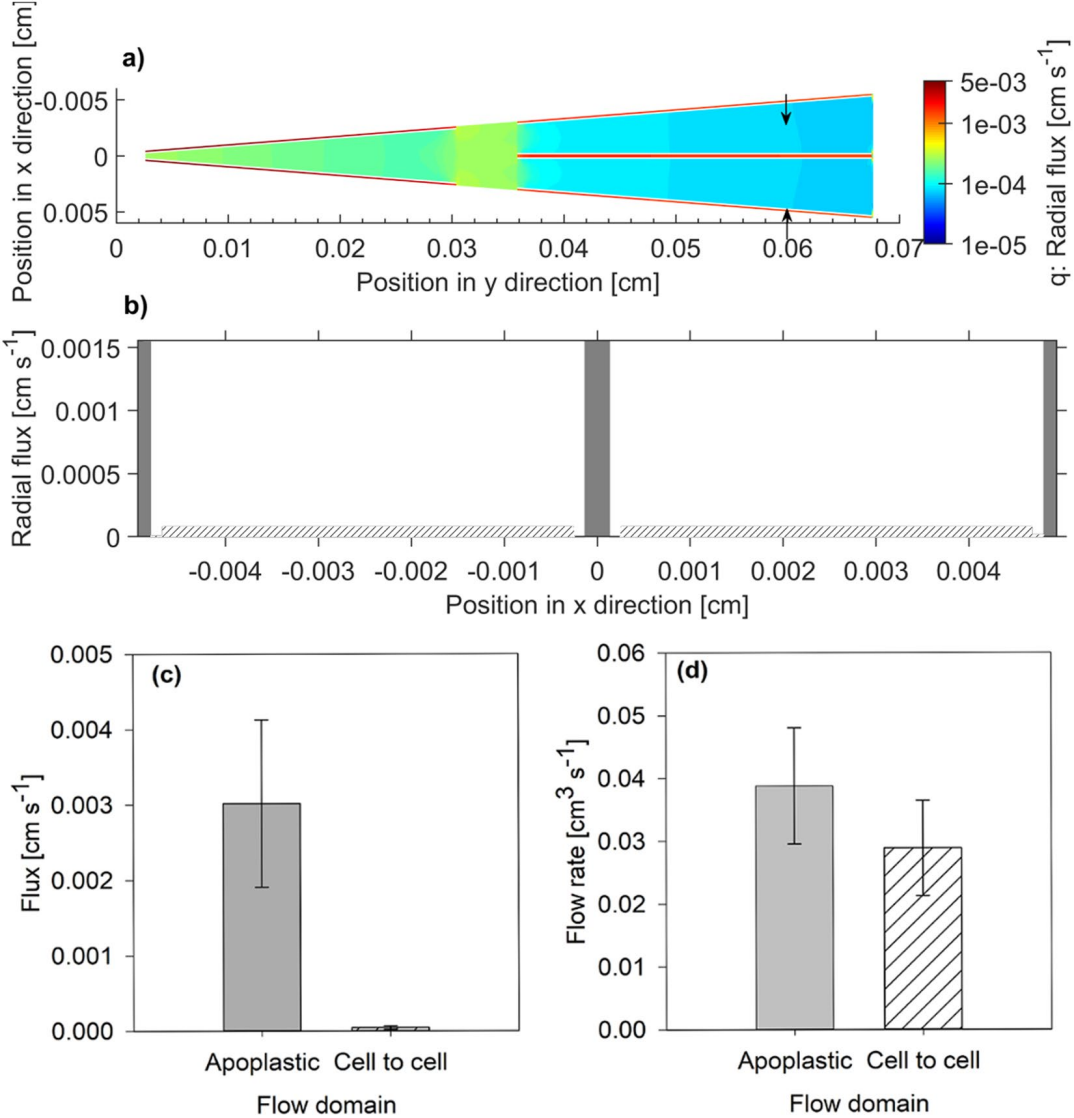
(**a**) Color mapped distribution of radial flux across the root tissue (*q*_*r*_, Eq. 10). (**b**) A transverse profile of radial flux in the cortex at a distance of 0.06 cm from the root center (position indicated by two arrows in subplot a). (**c**) Average flux across the apoplastic and cell-to-cell pathways. (**d**) The total flow of water across the apoplastic and cell-to-cell pathways (*Q*_*r,apo*_ and *Q*_*r,cell*_, Eq. 9). Note that in (**b**,**c**) the data are averaged across three plants and are referring to a position with a distance of 0.06 cm from the root center in the root cortex. The error bars show the standard deviation.

The solution of the inverse problem gave the spatial distribution of water flux across the root tissue (q_*r*_, Eq. (10), Fig. 4a). The radial flux [m s^−1^] was greater in the apoplastic pathway than in the cell-to-cell pathway. The profiles of radial flux at a distance of 0.06 cm from the root center are plotted in Fig. 4b. At this location, the radial flux of water through the apoplastic pathway was 104 ± 73 times higher in the apoplastic pathway than in the cell-to-cell pathway (Mean ± SD for n = 3) (Fig. 4c). This ratio was rather similar across the root tissue except at the endodermis where the apoplastic pathway was blocked^17–19^. Despite a higher radial flux through the apoplastic pathway, the overall contribution of this pathway in the transport of water (*Q*_*r,apo*_. Eq. 9) was rather similar in both pathways (Fig. 4d). At this selected location the apoplastic pathway contributed to 57 ± 8% (Mean ± SD for n = 3) of total water taken up by roots.

To simulate the transport of D_2_O across the root tissue three parameters were needed to describe the transport of D_2_O into the root via diffusion during nighttime and four parameters to describe the convective transport of D_2_O into the roots during the daytime (see Materials and Methods). It was further assumed that diffusion coefficients did not vary between day and night. We evaluated the sensitivity of the model to the parameters by changing the best-obtained parameters by a factor of 0.2 to 5 for the night measurement and by a factor of 0.1 to 10 for the day measurement and for each of the 25000 runs we calculated the predefined objective function (Eq. 14) (Supplementary Information Figs S3 and S4).

**Figure 5:**
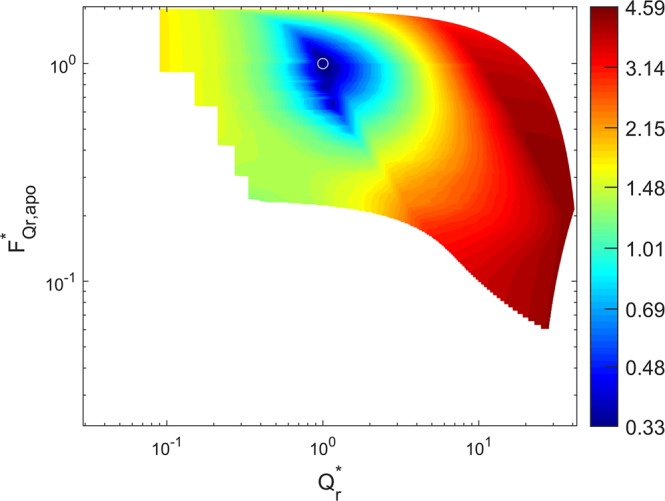
Sensitivity analysis of the model to the magnitude and spatial distribution of water fluxes across the root tissue. The hydraulic conductivity of different parts of the root tissue (apoplast, cell membrane, endodermis, and protoplast) was multiplied by a factor ranging from 0.1 and 10 and their effects on the objective function (Eq. 14), flow of water into the roots (*Q*_*r*_ = *Q*_*r,apo*_ + *Q*_*r,cell*_, Eq. 9), and the relative contribution of the apoplast to (*F*_*Qr,apo*_, Eq. 11) were calculated. The colormap shows the value of the objective function as a function of the estimate *Q*_*r*_ and *F*_*Qr,apo*_. The sensitivity analysis was performed around the optimal solution of fitted hydraulic conductivities. Two parameters were changed simultaneously while the others were kept constant. Note that the fluxes are shown here with a subscripted star which refers to the normalized values relative to their optimal fitted value.

A global minimum was reached with multiple sets of diffusion coefficients and hydraulic conductivities, indicating that the inverse problem can be solved with different sets of cell-scaled hydraulic properties of the root tissue (Figs S3 and S4). Although the model was not sensitive to all hydraulic parameters, it was sensitive to the water fluxes. The sensitivity analysis showed that a global minimum was reached around the total radial flow and the ratio of apoplastic and cell-to-cell water flow which gave the best fit with rather small uncertainty (Fig. 5). This proves that the model was sensitive to both, the total radial flow of water and the ratio between the apoplastic and the cell-to-cell flow. We conclude that the proposed method allows the estimation of the relative contribution of the apoplast and cell-to-cell pathways to the transport of water across the root cortex.

## Discussion

Our novel method combines experiments and modeling to quantify the fluxes of water across the root tissue. Rapid neutron tomography was used to *in-situ* trace the transport of D_2_O in roots. The imaged concentrations of D_2_O were inversely simulated to solve a diffusion-convection equation for the composite transport of water across the root tissue. The solution of the inverse problem gave the cell-scaled water fluxes. The novelty of this study was to quantify the spatial distribution of D_2_O over time in three dimensions and to estimate relative contributions of different pathways of water across root tissues. A recent method for *in-situ* quantification of water fluxes along the root system of transpiring plants^29,30^ was the starting point to quantify the relative importance of different pathways of water across the root tissue. The former method allowed to estimate the total radial flow, but it could not solve the relative importance of the apoplastic and cell-to-cell pathways. The main challenge was to push the limit of neutron tomography towards a much faster data acquisition while maintaining a high spatial resolution. This was successfully accomplished at the imaging station ICON at Paul Scherrer Institute. The imaging facilities allowed us to resolve the transport of D_2_O across the root tissue at a voxel size of 45 µm and with a tomography acquisition time of 30 s. Gradients in D_2_O concentration across the root tissue were visible and, by means of an inverse modeling approach, the contribution of the different pathways in the transport of water across the root tissue was quantified.

To quantify the flow field of water across the root tissue, we used a simplified domain consisting of a cell-to-cell pathway encapsulated by a thin layer representing the cell membrane and the remaining inner space representing the cell protoplast. The apoplastic pathway was considered as a flow domain between the cell membrane of neighboring cells. The cell-to-cell pathway was assumed to be a continuous flow domain interrupted at the endodermis, which had a cell membrane with lower specific permeability than the rest of the cells. Similarly, the apoplast was assumed to have a straight shape. In reality, the cell-to-cell pathway consists of several layers of cells, with water flowing from cell to cell by crossing two adjacent cell membranes or through plasmodesmata. Additionally, the apoplast is not straight but rather tortuous and with three-dimensional geometry. Explicitly solving the flow model in the exact cell geometry, including the plasmodesmata and the complex three-dimensional architecture of the apoplast, would introduce a large number of parameters that are not easily extractable. Therefore, to reduce the number of unknowns, we kept the flow domain as simple as possible, but complex enough to include the two pathways, their exchange and the role of the endodermis. Recently, Couvreur *et al*.^3^ developed a hydraulic model that computes the convective flow of water through walls, membranes, and plasmodesmata of each individual cell throughout a complete root cross section. Including the diffusive transport of D_2_O in such a model is a promising development of our method to reveal cell-scaled hydraulic properties of roots and the water fluxes across the root tissue including its anatomical complexities. Another important assumption of our model is that the diffusion coefficients did not vary between day and night. However, the diffusion coefficient of the membrane might change in response to varying aquaporin activity. Additionally, water flow is likely to increase the dispersivity of D_2_O through the apoplast, resulting in a greater apparent diffusivity of the apoplast. As the apoplast is more diffusive than the endodermis and as convection is dominant through the apoplast (during daytime), it is unlikely that assuming constant diffusivity in the apoplast impacts our result. Independent measurements of water during the experiments might allow for releasing the assumption that the diffusion coefficients are constant and for studying how these coefficients vary over time and soil and climate conditions.

Our results showed that the apoplast, despite occupying a small fraction of the root cross section (ca. 5%), contributes to half of the transport of water flowing across the cortex (57 ± 8% of total water taken up by the roots). This is explained by the higher hydraulic conductivity of the apoplast compared to the cell-to-cell pathway. This result is in agreement with the literature. For instance, apoplastic conductance of cherry seedlings was 57% of the total root conductance, suggesting that its contribution in overall root water uptake would be 57%^26^. A similar result was reported for lupin roots grown in soil by Bramley *et al*.^15^, who reported that in a narrow-leafed lupin water moves predominantly through the apoplastic pathway. However, those measurements refer to the contribution of the apoplast to the total hydraulic conductance, while our data refer to the flow rates across both apoplastic and cell-to-cell pathways. So, this information is not comparable in a straightforward way.

The hydraulic model coupled with the diffusion-convection equation was needed to solve the inverse problem and estimate the distribution of water fluxes across the root tissue. The model required a large number of parameters and to some of these parameters, such as the hydraulic conductivities, the model was not sensitive. (Supplementary Information Figs S3 and S4). Additionally, the estimated values of these parameters, in particular, the hydraulic conductivities of the different pathways, are affected by the simplified geometry of the flow domain. Therefore, the estimation of the cell-scale hydraulic conductivities and diffusion coefficients has a high degree of uncertainty. However, independently from these parameters, the inverse problem was sensitive to the total flow of water and to its spatial distribution. The sensitivity analysis showed that both, the total radial flow and the relative importance of apoplast and cell-to-cell, were estimated with high accuracy. Therefore, compared to the uncertainty in the cellular-scale hydraulic and diffusion parameters, the estimation of water fluxes is accurate.

In conclusion, the proposed method allows to non-invasively quantify the fluxes of water across the root tissue and the relative importance of their pathways. We found that fluxes are not uniform, with the flux in the apoplast being much higher than across the cell-to-cell, and with the endodermis partly or completely blocking the apoplastic pathway. This method offers new opportunities to answer long-standing open questions: for instance, it could be used to test whether and to what extent (i) root permeability varies with soil drying and transpiration, and (ii) varying root permeabilities is an adaptive root trait in response to water stress. Such alterations of root permeabilities might take place at the endodermis, due to suberization, or due to varying aquaporin activity of all root cells or of some of them. The information provided by a rapid neutron tomography could help to answer such open questions in root-soil water relations.

## Materials and Methods

### Plant and soil preparation

We grew six lupines (*Lupinus albus* L. cv. Feodora) in graphite cylindrical containers (a diameter of 27 mm and a length of 100 mm) filled with a sandy soil collected from an Ap horizon. The soil consisted of 73% sand, 18% silt, and 9% clay. The containers were filled vertically by pouring the dry soil through a 2 mm sieve, which resulted in a homogeneous packing. The seeds were soaked for 10 minutes in 10% H_2_O_2_ solution, were thoroughly washed, and then were let germinate on filter paper in a 10 mM L^−1^ CaSO_4_ solution for 48 h. One seedling was sown in each container at a depth of 1 cm. The plants were kept in a climate chamber under controlled conditions during the whole growth period: day-night temperature of 24–19 °C, the daily light cycle of 14 hours, the light intensity of 500 µmol m^−2^ s^−1^ at the top of the canopy, and relative humidity of 60%. When the plants were three days old, a one-centimeter layer of quartz gravel (grain diameter of 1.7–2 mm) was added on the top of the soil to minimize evaporation. Plants were irrigated by capillary rise every fourth day.

The neutron tomography measurements started when the plants were 12 days old. Prior to the measurement, plants were placed in the imaging station ICON at the Paul Scherrer Institute and transpiration was monitored gravimetrically by weighing the samples at two different times with an interval of three hours. Daytime transpiration was 0.65 ± 0.13 cm^3^ h^−1^ and it was negligible at night-time. Three plants were tomographed at daytime and the other three at nighttime.

### Neutron tomography

Sequences of neutron tomographies allow for imaging the spatiotemporal distribution of water in soil and plants^34,35^. A parallel neutron beam crosses the sample while the sample is continuously rotated at constant angular velocity while the neutron detector acquires projections at a given frame rate. The transmitted neutron beams at each angle carry information about the composition and thickness of the sample. 3D volumetric information is obtained by reconstructing the projections taken at different angular views using the filtered back projection algorithm^36^.

Neutron tomography was performed at the beamline ICON at Paul Scherrer Institute (PSI), Villigen, Switzerland. The sample was fixed on a rotational stage whose axis of rotation was placed at about 20 mm upstream the scintillator screen. The rotational stage together with the negligible readout time of sCMOS detector available at ICON beamline allowed for the application of a high temporal resolution on-the-fly tomography. The sCMOS detector was set to acquire images of 1200 pixels in width and 2560 pixels in height, the exposure time was set to be 0.1667s (6 frames/s), the rotation rate of the sample stage was set at 0.5°/s and the proton current at the SINQ target was about 1.43 mA, which corresponds to a neutron flux of approximately 1.25 × 10^7^ neutrons cm^−2^ s^−1^ at the sample position. The temporal resolution of the series of neutron tomographies (from the angular view of 0° to 179°) was 30 s. While the nominal pixel size of the acquired images corresponded to 45 µm, the spatial resolution in 2D is about 200 μm/line pair as assessed visually from the image of PSI test pattern Gd-based Siemens star^37^. The detector system allowed an on-the-fly tomography consisting of 7000 projections. The experiments were performed in such a way that 762 projections were acquired before D_2_O injection. Then, D_2_O was injected into the sample from the top using a remotely controlled *in-situ* titration system^38^ while the sample was simultaneously tomographed.

### Image processing

A set of parallel beam projections obtained from angular views in the interval 0° to 179° were reconstructed into a 3D volume using a filtered back projection algorithm in Matlab 2015b. Prior to the reconstruction, neutron radiographs were normalized using the Beer-Lambert law for flat field (I_0_), dark current (I_dc_) and beam variation over time as follows

1 $$\tau =-\log (\frac{I-{I}_{dc}}{{I}_{0}-{I}_{dc}}\times \frac{{D}_{0}}{D})$$

where *τ* is the optical thickness (sample thickness × attenuation coefficient) [−], *I* is the grey level proportional to the attenuated neutron beam [# neutrons], *I*_0_ is the grey level proportional to the incident neutron beam [# neutrons], D and D_0_ are scalar values proportional to the neutron dose. They are estimated as the average value from an image region outside the sample projection through all the radiographs and flat field, respectively.

After normalization, the projections were rearranged into sinograms, i.e. images containing the information required to a reconstruct a single slice. The resulted sinograms were first filtered to remove ring artifacts^39^ and then back projected using the *iradon* function in Matlab to reconstruct transverse slices of the sample. The *iradon* function in Matlab assumes that the center of rotation is the center point of the projections. This function was modified to take a customized center of rotation with a subpixel precision (0.01 × spatial resolution of 2D images). The center of rotation with a subpixel precision was determined based on integral of absolute values of a reconstructed slice proposed by Donath *et al*.^40^.

The reconstructed voxels represent the average attenuation coefficient of the materials present in the sample sub-volume at the position of the voxel. For voxels containing roots, this value can be described in terms of volumetric fractions of H_2_O, D_2_O, and soil as

2 $${\mu }_{tomo}(t)={F}_{H2O}(t){\mu }_{H2O}+{F}_{D2O}(t){\mu }_{D2O}+{F}_{roottissue}{\mu }_{roottissue}$$

where *μ*_*tomo*_(*t*) is the voxel-wise neutron attenuation coefficient at time t in the tomograms [cm^−1^], *μ*_*H*2*O*_, *μ*_*D*2*O*_ and *μ*_*root tissue*_ are the linear neutron attenuation coefficient [cm^−1^] of water, D_2_O, and root tissue (dry biomass of root), respectively, and F are their volumetric fractions [−]. Note that the sum of the volumetric fraction of the different phases is equal to one. Here it assumed that the contribution of dry root tissue in neutron attenuation is negligible compared to the contribution of the liquid phase (volumetric water content of the root is ca. 90%). The neutron attenuation coefficient describes the probability of neutron interactions with the materials per unit of thickness and was determined by neutron radiography of known thickness of each component (H_2_O, D_2_O, and root tissue).

It is assumed that volumetric liquid content of the root tissue did not change after immersion in D_2_O. Then the voxel-wise concentration of D_2_O in the voxel containing root can be calculated as

3 $$C=\frac{{F}_{D2O,root}}{{F}_{liquid,root}}$$

where

4 $${F}_{liquid,root}=\frac{{\mu }_{tomo}(t=0)}{{\mu }_{H2O}}$$

5 $${F}_{D2O,root}=\frac{{\mu }_{tomo}(t)-{\mu }_{tomo}(t=0)}{{\mu }_{D2O}-{\mu }_{H2O}}$$

where *C* is the concentration of D_2_O in roots (fraction of D_2_O to total water), *F*_*liquid,root*_ is the volumetric fraction of liquid (H_2_O + D_2_O) in the voxels containing root, *F*_*D*2*O*_,_*root*_ is the volumetric fraction of D_2_O in the voxels containing root, *t* refers to the time after D_2_O injection and *t* = 0 is the time when D_2_O was injected. Equation 3 is only valid for calculation of D_2_O concentration in the roots and its assumption (volumetric liquid content of the root tissue did not change after D_2_O injection) is not be valid in soil. The concentration of D_2_O in soil was calculated from the neutron radiographs of the entire sample following the approach presented in Zarebanadkouki *et al*. (2013).

### Modeling of D_2_O transport

The radial transport of D_2_O into the root is modeled using a diffusion-convection equation where diffusion depends on the concentration gradient across the root tissue and the diffusional permeability of the root tissue, and convection depends on the gradient in water potential between xylem and soil and the permeability of the root tissue^29,30^. The equation can be written as:

6 $$\theta \frac{\partial C}{\partial t}=\nabla (D\Delta C)-\nabla (qc)$$

where *θ* is the volumetric water content [cm^3^ cm^−3^], C is the concentration of D_2_O [cm^3^ cm^−3^], D is diffusion coefficient [cm^2^ s^−1^] of the root tissue, q is the flux of water [cm s^−1^], ∇ is the divergence in space and Δ is the gradient in space. The concentration of D_2_O over time was quantified through the neutron tomograms based on Eq. 3. The flux of water [q, cm s^−1^] can be computed as

7 $$q=K\Delta h$$

where *K* is the hydraulic conductivity across the root tissue [cm s^−1^] and *h* the is water potential expressed in centimeter heads [cm]. Under steady-state conditions, the profile of water potential across the root tissue is computed by solving the equation below

8 $$0=-\,\nabla (K\Delta h)$$

With a known boundary condition (water potential in the soil and in the xylem) Eq. 7 gives the velocity of water across the root tissue (*q*) in the apoplastic and cell-to-cell pathways. The overall contribution of each pathway in root water uptake [cm^3^ s^−1^] depends not only on the radial flux of water, *q*_*r*_, through each pathway but also on their volumetric fraction. The total flow of water in each pathway was calculated as follows

9 $$\begin{array}{rcl}{Q}_{r,apo} & = & 2\pi r\overline{{q}_{r,apo}}{\omega }_{apo}\\ {Q}_{r,cell} & = & 2\pi r\overline{{q}_{r,cell}}{\omega }_{cell}\end{array}$$

where *Q*_*r*,*apo*_ and *Q*_*r,cell*_ are the total flow of water through the apoplastic and the cell-to-cell pathway per unit of root length, respectively [cm^3^ s^−1^ cm^−1^], *r* is the distance from the root center [r = 0.06 cm], $\overline{{q}_{r,apo}}$ and $\overline{{q}_{r,cell}}$ are the average radial flux [cm s^−1^] through the apoplastic and the cell-to-cell pathways, and *ω*_*apo*_ and *ω*_*cell*_ are the volumetric fractions of the apoplastic and cell-to-cell pathways. To do so, *q*_*r*_ across the root tissue was calculated as follows

10 $${q}_{r}=\sqrt{{(K\frac{\partial h}{\partial x})}^{2}+{(K\frac{\partial h}{\partial z})}^{2}}$$

Here, *x* and z are the cartesian coordinates across the root tissue. The overall contribution of each pathway in the total flow of water was calculated as

11 $$\begin{array}{rcl}{F}_{Qr,apo} & = & \frac{{Q}_{r,apo}}{{Q}_{r,tot}}\\ {F}_{Qr,cell} & = & \frac{{Q}_{r,cell}}{{Q}_{r,tot}}\end{array}$$

where *Q*_*r*,*tot*_ is the total flow of water across the root tissue and it is equal to *Q*_*r*,*apo*_ + *Q*_*r,cell*_.

### Model implementation and parameterization

For a quantitative description of water flow across the root tissue, we conceptualized the complex structure of the root tissue as shown in Fig. 3. The root cortex was represented as two cell-to-cell pathways that were surrounded by three apoplastic pathways in their longitudinal direction. Two apoplastic pathways were located on the sides of each cell-to-cell pathway and one apoplastic pathway was located between them. The middle apoplastic pathways had a double thickness as the ones on the sides taking into account that the apoplastic pathways on the sides are shared between their surrounding cell-to-cell pathways outside of the simulated domain. The apoplastic domain was assumed to occupy 5% of the root volume. Note that for computational reasons we had to take this volumetric fraction slightly bigger than the value reported by Fritz and Ehwald^19^ (ca. 3% for maize root). Each cell-to-cell pathway was encapsulated by a thin region (with a thickness of 1.3 µm) representing the cell membrane and the remaining inner space representing the cell protoplast and cell plasmodesmata. The apoplastic pathway was fully interrupted at a distance of 0.45 times the root radius from the root center (root radii of ca. 0.6–0.7 mm, obtained from root cross-section) by a distinct layer of cells (with the thickness of one cell) representing the root endodermis. Note that the endodermis was assumed to be one cell in thickness, surrounded by two membranes on its sides and the inner space of this cell was assumed to be similar to the protoplast of other root cells. The stele was represented by one cell-to-cell pathway that was encapsulated by the cell membrane and surrounded by two apoplastic pathways in its side in the longitudinal direction. The flow domain arrived till the xylem vessels.

The flow domain and cellular scale hydraulic information were combined to build a finite element model for simulating the transport of D_2_O across the root tissue. The flow domain was discretized into 2000 elements of varying size. Eq. 6 was numerically solved using an implicit finite element method in Matlab. To numerically solve this equation, we imposed Dirichlet boundary condition (known D_2_O concentration shown as Supplementary Information Fig. S5) at the root surface and Neumann boundary condition (zero flux) in the xylem. The diffusion coefficients [cm^2^ s^−1^] of the following domains were the parameters needed to solve Eq. 6: the apoplastic pathway (*D*_*apoplast*_), the membrane of root cells (*D*_*cell membrane*_), the membrane of endodermis (*D*_*endormis*_), and the cell protoplast (*D*_*protoplast*_). To describe the convective transport of D_2_O across the root tissue, the flow field was first reconstructed by numerically solving Eqs 7 and 8. These equations were solved by imposing Dirichlet boundary condition (known water potential) in soil and xylem. The hydraulic conductivities [cm s^−1^] of the following flow domains were needed: apoplastic pathway (*K*_*apoplast*_), the membrane of root cells (*K*_*cell membrane*_), the membrane of endodermis (*K*_*endodermis*_), and the cell protoplast (K_*protoplast*_). Note that the diffusion coefficients and hydraulic conductivities at the root-soil interface were scaled by the volumetric soil water content. Across the root tissue, the volumetric water content is around 100% while it was around 20% in the soil suggesting that only 20% of the root area will be available for D_2_O to flow into the root. For the membranes, the diffusion coefficients are related to the diffusional permeabilities (*Pd* with a unit of cm s^−1^) as following:

12 $$\begin{array}{rcl}P{d}_{cellmembrane} & = & \frac{{D}_{cellmembrane}}{{d}_{cellmembrane}}\\ P{d}_{endorermis} & = & \frac{{D}_{endodermis}}{{d}_{endodermis}}\end{array}$$

where *d*_*cell membrane*_ and *d*_*endodemris*_ are the thicknesses of the membrane of root cells and of the endodermis [cm], respectively. The hydraulic permeabilities of the membranes were also defined as

13 $$\begin{array}{rcl}L{p}_{cellmembrane} & = & \frac{{K}_{cellmembrane}}{{d}_{cellmembrane}}\\ L{p}_{endodermis} & = & \frac{{K}_{endodermis}}{{d}_{endodermis}}\end{array}$$

where is *Lp* is the membrane hydraulic permeability [cm cm^−1^ s^−1^ which is equivalent to 10^4^ cm MPa^−1^ s^−1^].

The profiles of D_2_O concentration during nighttime were used to estimate the cell scaled diffusion coefficients of *D*_*cell membrane*_, *D*_*endodermis*,_ and *D*_*protoplast*_ by inversely solving Eq. 6 and adjusting these diffusion coefficients to best reproduce the measurements. To reduce the number of unknowns, *D*_*apoplast*_ was assumed to be one-sixth of the self-diffusion coefficient of D_2_O (*D*_0_ = 2.27 × 10^−5^ cm^2^ s^−1^ ^41^). Published values of diffusion coefficients for different solutes in the apoplast range from 1/5 to 1/60 of the diffusion in pure water^21,42–44^. We took a value close to the highest one (1/6) because D_2_O is a neutral molecule with a rather similar molecular weight compared to normal water. Lower values are expected for bigger and charged molecules. Note that we assumed that the change in soil water potential (inducing a convective transport of D_2_O across the root tissue) after D_2_O injection was negligible. Assuming that diffusion coefficients were constant during day and night, the profiles of D_2_O concentration during daytime were used to estimate the hydraulic conductivities of *K*_*apoplast*_, *K*_*cell membran*,_ *K*_*endodemris*_ and *K*_*protoplast*_ by inversely solving the Eq. 6 and adjusting these conductivities to best reproduce the measurements. To solve the equation, the xylem and soil water potentials were needed. The water potential in the soil was ca. −100 cm (based on the measured water content and water retention curve). The xylem water potential was measured in plants grown in the same conditions and imposed to the same transpiration rate using the Scholander bomb^45^ and it was 4000 cm. At the peak of the transpiration, plant shoots were carefully cut with a sharp razor blade and placed in Scholander bomb exposing the cut end to the atmosphere. Then the pressure at which a drop of water was observed at the cut end of the stem was taken as the leaf water potential.

The inverse problem was solved by minimizing a predefined objective function using the ‘global optimization toolbox’ in Matlab (‘pattern search’ algorithm). The ‘pattern search’ algorithm finds the global minimum of a pre-defined objective function. This solver has the advantage of accepting lower and upper pre-defined boundaries for the solution. It also allows linear and non-linear constraints to the solution. The objective function (*Obj*) to be minimized was defined as the root mean square of relative differences between measured and simulated D_2_O concentration at different locations across the root tissue at different times.

14 $$Obj=\Vert \frac{{({C}_{i,j}^{mes}-{C}_{i,j}^{sim})}^{2}}{{({C}_{i,j}^{mes})}^{2}}\Vert $$

where $\Vert \cdot \Vert$ refer to the norm of a matrix, ${C}_{i,j}^{mes}$and ${C}_{i,j}^{sim}$are the measured and simulated concentration of D_2_O at location *i* and time *j*, respectively. Here *i* refers to the distance from the root surface and *j* to the time after immersion in D_2_O.

Before any effort to solve an inverse problem, it is very important to find out whether the pre-defined objective function is sensitive to the parameters to be optimized. A sensitivity analysis was performed around the optimal solution of fitted diffusion coefficients and hydraulic conductivities. A local sensitivity analysis was carried out by simultaneously varying two selected parameters confirming the sensitivity of the model to its parameters.

## Supplementary information

Suplimentary infromation
