# Supplementary material for: Root water uptake and its pathways across the root: quantification at the cellular scale
Source: Sci Rep. 2019 Sep 10;9:12979. doi: 10.1038/s41598-019-49528-9 (PMC6737181; doi:10.1038/s41598-019-49528-9)
Supplement: Supplementary file 1 — Suplimentary infromation [file 41598_2019_49528_MOESM1_ESM.docx]

**Root water uptake and its pathways across the root: quantification at the cellular scale**

Mohsen Zarebanadkouki^*1^, Pavel Trtik^2^, Faisal Hayat^1^, Andrea Carminati^1^, Anders Kaestner*^2^

^1^ Chair of Soil Physics, University of Bayreuth, Germany

^2^ Laboratory for Neutron Scattering and Imaging, Paul Scherrer Institut, Villigen, Switzerland

^*^ Corresponding authors

Mohsen Zarebanadkouki, [mohsen.zarebanadkouki@uni-bayreuth.de](mailto:mohsen.zarebanadkouki@uni-bayreuth.de)

Anders Kaestner, [Anders.kaestner@psi.ch](mailto:Anders.kaestner@psi.ch)

**List of suplimentary infromation:**

Fig. S1: Concentration of D_2_O at different times after D_2_O injection during nighttime and daytime.

Fig. S2: Estimated diffusion coefficient (D), diffusional permeability (Pd), hydraulic conductivity (K) and hydraulic permeability (Lp) of different parts of the root tissue.

Fig. S3: Sensitivity analyses of the model parameters for the simulation of diffusion across the root tissue.

Fig. S4: Sensitivity analyses of the model parameters for the simulation of water flow (convection) across the root tissue.

Fig. S5: Profile of D_2_O concentration at the root surface in the soil during both day and night measurement.


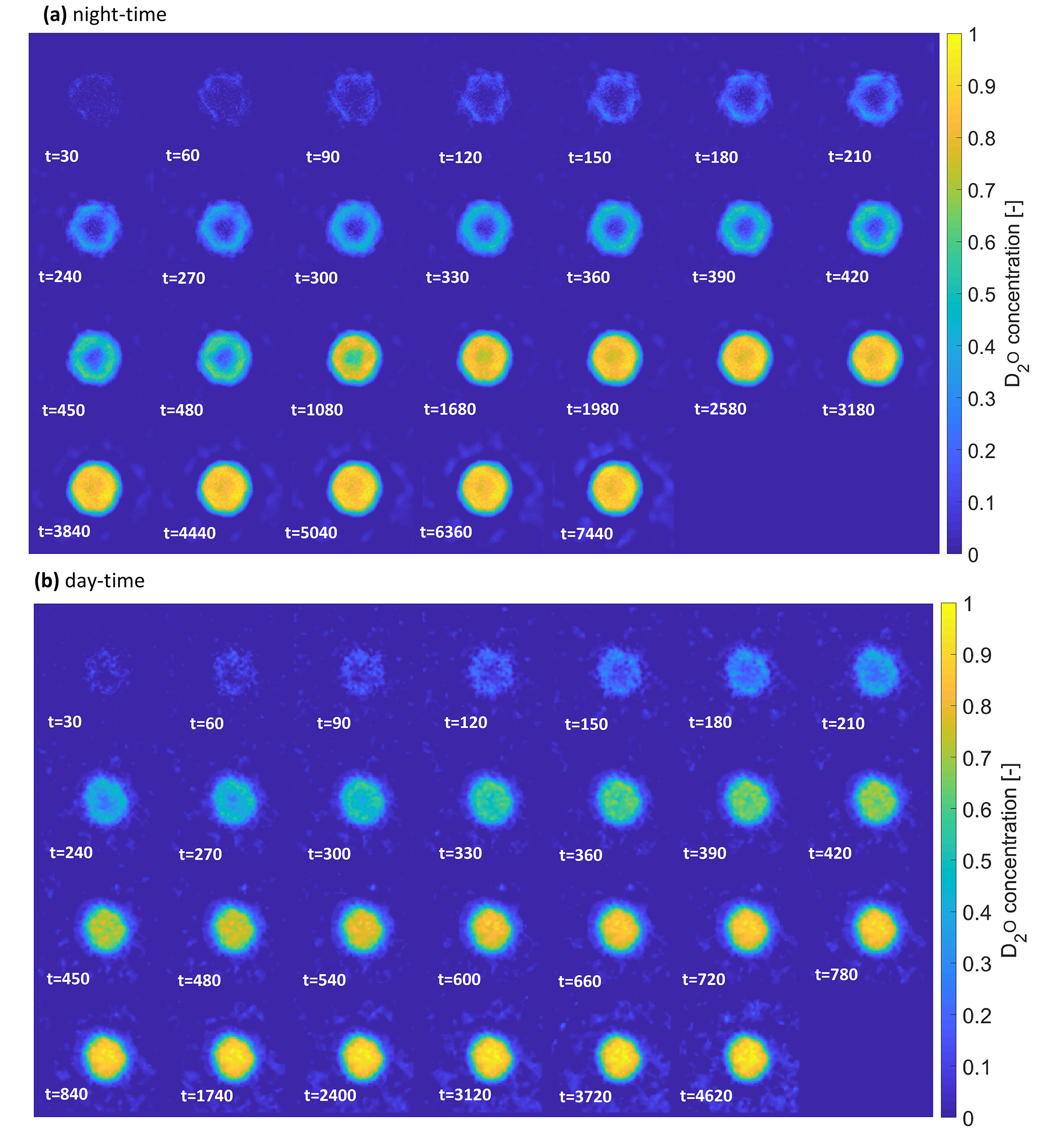


Fig. S1: Concentration of D_2_O at different times after D_2_O injection during nighttime (a) and daytime (b). The given time (t in seconds) refers to the time after D_2_O injection. These images show that the transport of D_2_O across the root tissue was faster during the daytime than nighttime. At nighttime, the transport was remarkably slowed down by the presence of endodermis.


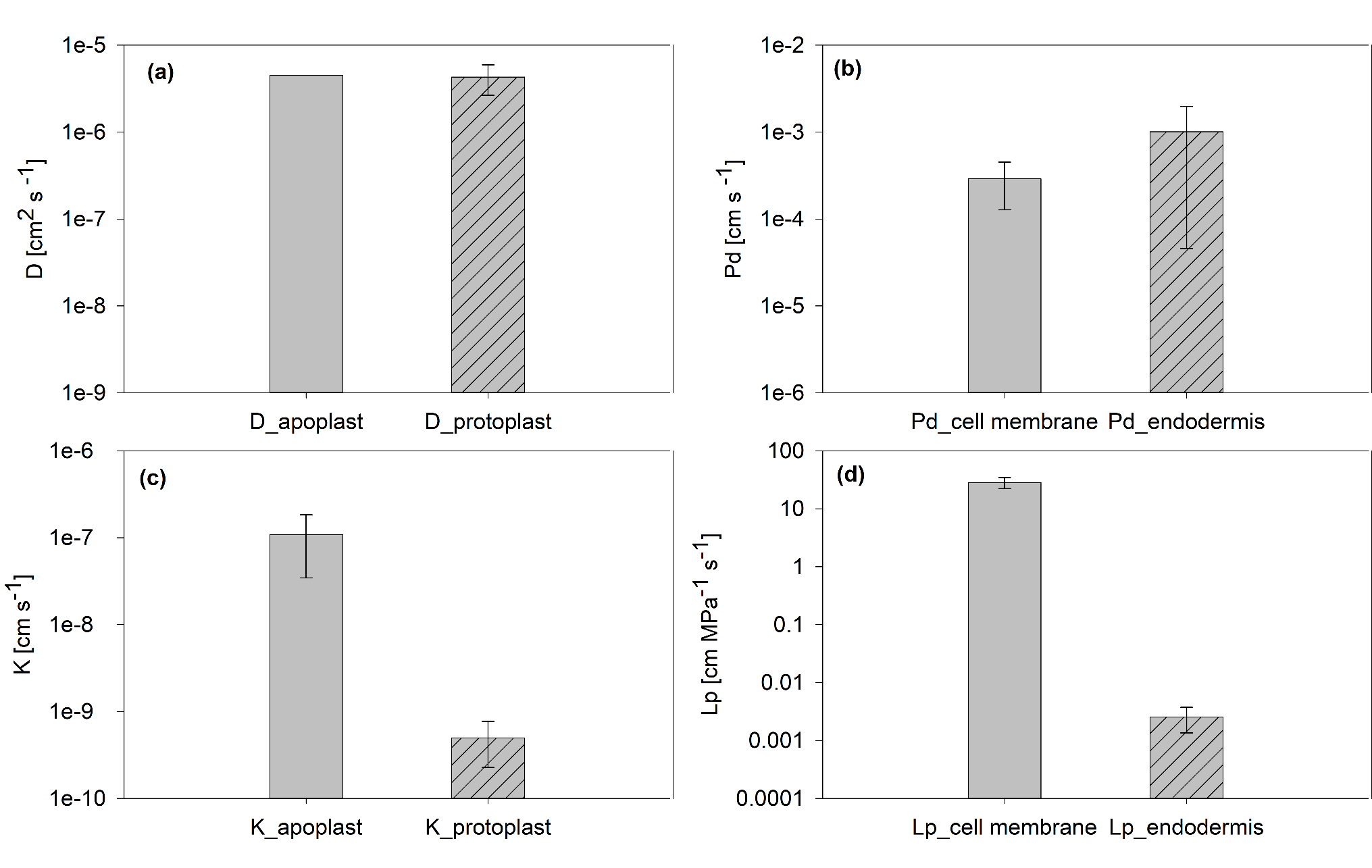


Fig. S2: (a-b) Diffusion coefficient (D) and diffusional permeability (Pd) of root tissue obtained from the simulation of night measurement. (c-d) Hydraulic conductivity (K) and hydraulic permeability (Lp) of different parts of the root tissue obtained from day measurements. The values are average of three plants and error bars show the standard deviations. Note that D of apoplast was assumed to be constant.


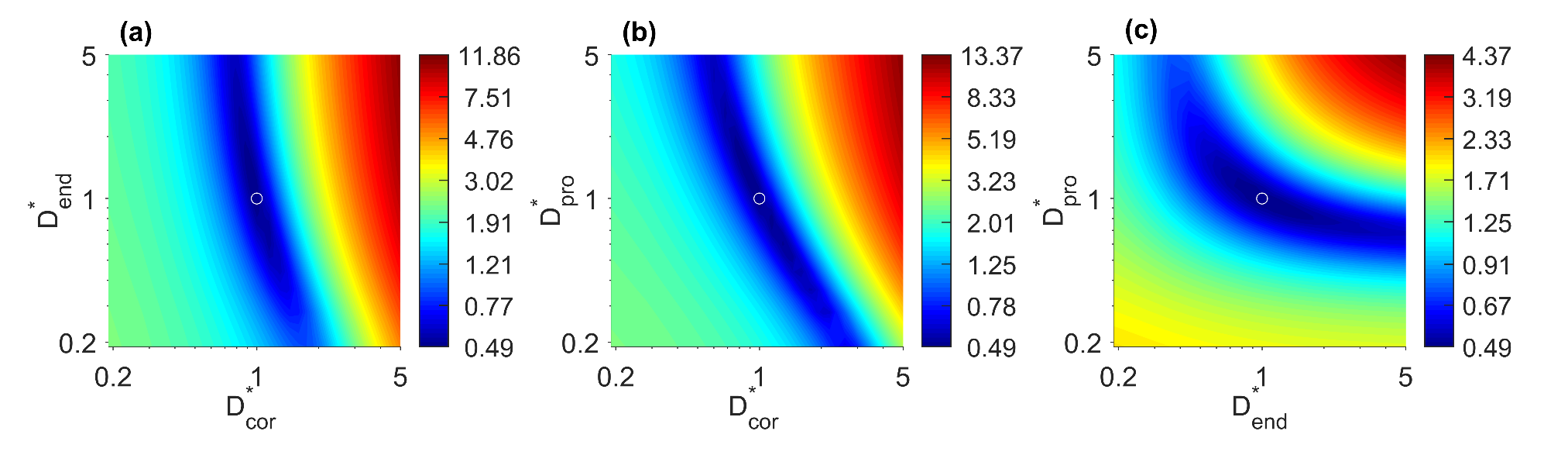


Fig. S3: Sensitivity analyses of the model parameters for the simulation of diffusion across the root tissue. Model parameters are changed by a factor of 5 and the resulting predefined objective functions (Obj, Eq.14) are shown as color-mapped images . The sensitivity analysis was performed around the optimal solution of fitted diffusion coefficients. Two parameters were changed simultaneously while the others were kept constant. Note that each model parameter is shown here with a subscripted star which refers to the normalized value of each parameter by its optimal fitted value.


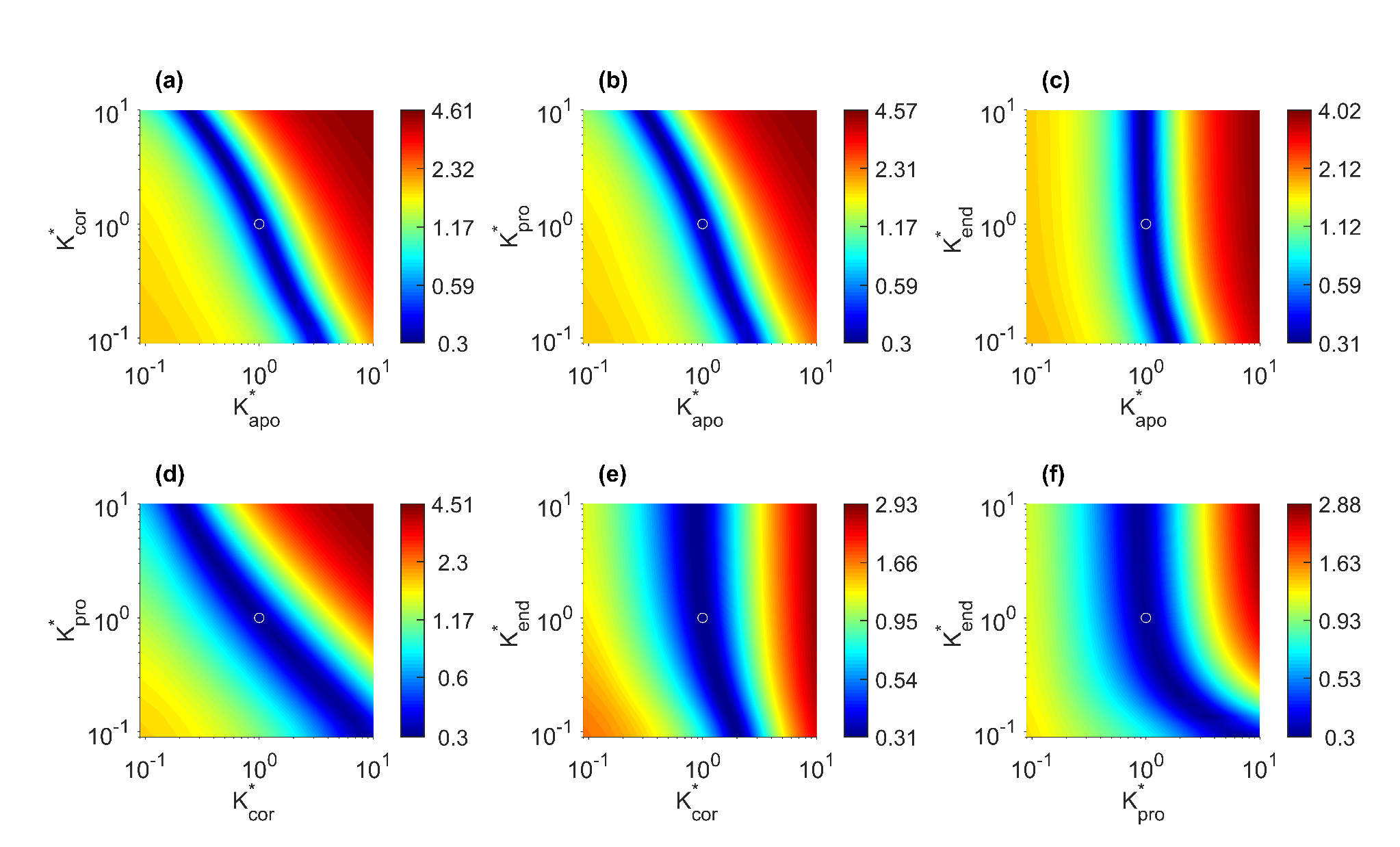


Fig. S4: Sensitivity analyses of the model parameters for the simulation of water flow (convection) across the root tissue. Model parameters are changed by a factor of 10 and the resulting predefined objective functions (Obj, Eq.14) are shown as color-mapped images. The sensitivity analysis was performed around the optimal solution of fitted hydraulic conductivities. Two parameters were changed simultaneously while the others were kept constant. Note that each model parameter is shown here with a subscripted star which refers to the normalized value of each parameter by its optimal fitted value.


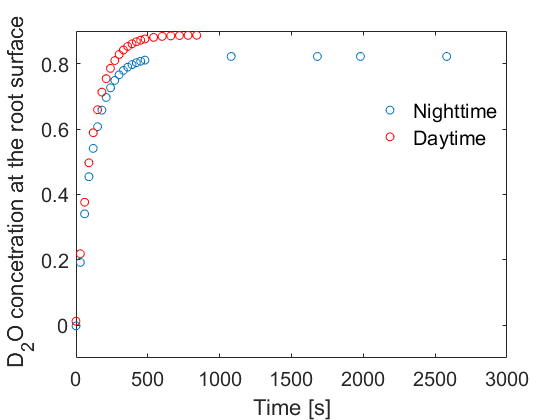


Fig. S5: Profiles of D_2_O concentration in the soil next to the root surface during day and night measurement. Note the profile of D_2_O concentration were smoothed to obtain more stability during numerical simulations.
